# A novel extracellular flux assay workflow uncovers impaired sciatic nerve mitochondrial respiration in diabetic db/db mice

**DOI:** 10.1186/s12964-026-02667-9

**Published:** 2026-01-21

**Authors:** Sebastian Sill, D. Margriet Ouwens, Fariba Zivehe, Sonja Hartwig, Stefan Lehr, Gidon J. Bönhof, Michael Roden, Hadi Al-Hasani, Alexandra Chadt, Alexander Strom

**Affiliations:** 1 Institute for Clinical Biochemistry and Pathobiochemistry, Medical Faculty, German Diabetes Center (DDZ), Leibniz Center for Diabetes Research at Heinrich Heine University, Düsseldorf, Germany; 2 German Center for Diabetes Research (DZD e.V.), Partner Düsseldorf, Neuherberg, Germany; 3 Institute for Clinical Diabetology, German Diabetes Center (DDZ), Leibniz Center for Diabetes Research at Heinrich Heine University, Düsseldorf, Germany; 4 Department of Endocrinology and Diabetology, Medical Faculty and University Hospital, Heinrich-Heine-University, Düsseldorf, Germany

**Keywords:** Diabetic neuropathy, Sciatic nerve, Mitochondrial bioenergetics, Extracellular flux analysis, Db/db mouse

## Abstract

Abnormal mitochondrial function contributes to the development of diabetic neuropathy by inducing oxidative stress and altering energy metabolism, ultimately leading to neuronal damage. However, direct and reproducible assessment of mitochondrial bioenergetics in peripheral nerves remains technically challenging. Here, we describe an optimized ex vivo approach for measuring mitochondrial respiration in isolated murine sciatic nerve segments using extracellular flux analysis. Although extracellular flux-based assays have previously been applied to nerve tissue, we introduce key methodological refinements, including optimized tissue preparation, assay conditions, and injection parameters, to achieve stable and reproducible mitochondrial responses. The innovations of our work include the use of mass spectrometry-acquired mitochondrial protein abundances (MitoCarta 3.0) for normalization of the extracellular flux data as well as the application of new computational algorithms that enable straightforward evaluation of mitochondrial toxicity, uncoupling, and alterations in oxidative phosphorylation. Using this workflow, we show that sciatic nerves from diabetic db/db mice exhibit impaired mitochondrial respiration compared to lean C57BLKS/J controls, characterized by reduced basal and maximal respiration, lower ATP-linked respiration, and decreased proton leak. Our approach provides a robust platform for studying mitochondrial bioenergetics in peripheral nerves and offers a scalable tool for evaluating therapeutic interventions in diabetic neuropathy and related disorders.

**Supplementary Information:**

The online version contains supplementary material available at 10.1186/s12964-026-02667-9.

## Introduction

Neurological disorders represent a leading cause of global disease burden, with rising disability-adjusted life years (DALYs), underscoring the urgent need for improved strategies in prevention and treatment [1]. Diabetic polyneuropathy is a debilitating complication of diabetes mellitus that affects nearly half of the individuals with chronic hyperglycemia [2] and ranks among the ten neurological disorders with the highest age-standardized DALYs [1]. Diabetic sensorimotor polyneuropathy (DSPN) represents the most frequent clinical manifestation of diabetic polyneuropathy. Current approaches to treat DSPN remain insufficient to prevent disease progression or to reverse established nerve injury. This highlights the need for early detection and further mechanistic research [3]. DSPN is characterized by progressive sensory and motor dysfunction resulting from complex metabolic and bioenergetic disturbances that impair nerve function [4, 5]. One of the key pathogenic mechanisms underlying DSPN involves changes in mitochondrial function in the peripheral nerve system, particularly the sciatic nerve, the largest motor and sensory nerve of the leg [6, 7]. These changes include reduced electron transport chain activity [8], diminished ATP production [9], a loss of mitochondrial membrane potential [10], excessive generation of reactive oxygen species [11], disrupted calcium handling [11], and inefficient removal of damaged mitochondria [12], all of which compromise neuronal energy supply and promote oxidative injury.

Changes in mitochondrial functionality play a critical role in the onset and progression of DSPN due to the high metabolic energy demands of neurons [13]. Thus far, it has been technically challenging to evaluate mitochondrial function in peripheral nerves due to their small size and metabolic heterogeneity [14]. High-resolution respirometry using the Oroboros O2k system is widely considered as the gold standard for detailed assessment of mitochondrial respiration [9, 15]. However, its throughput is limited, as only two samples can be measured simultaneously. Seahorse-based extracellular flux assays overcome this limitation by performing assays in 24- or 96-well plates, thereby allowing the simultaneous analysis of multiple replicates, as well as the inclusion of positive and negative reference samples for quality control and quantifications. These characteristics make the Seahorse technology the preferred method for larger studies, such as mechanistic and intervention studies.

Previous reports have already applied extracellular flux analysis to measure determinants of glycolytic and mitochondrial function in sciatic nerves [14, 16–18]. The current study aimed at improving these protocols by optimization of both the technical and analytical part of the assay, including the introduction of novel metrics to quantitate determinants of mitochondrial function in peripheral sciatic nerves. Refining the assay conditions, minimizing the variability by analysing multiple defined-length sciatic nerve segments and incorporating novel metrics may improve the depth and reliability of mitochondrial activity assessment, thus overcoming limitations observed in previous reports. To do so, we compared mitochondrial function using the conventional mitostress parameters as well as a newly developed algorithm in sciatic nerves isolated from control BKS and diabetic db/db mice. The db/db mice are a well-established model of type 2 diabetes, characterized by sustained hyperglycemia and progressive peripheral neuropathy [19]. Application of the refined methodology provided a more robust and reproducible characterization of alterations in mitochondrial function using high-resolution respirometry, thereby contributing to developing targeted therapeutic strategies to restore nerve energy metabolism and prevent neuropathic complications [8].

## Methods

### Chemicals, materials and buffers

Chemicals and materials are listed in Supplementary Tables 1 and buffer composition is detailed in Supplementary Table 2.

### Animal experiments

All animal procedures were performed in accordance with the German Animal Welfare Act and approved by the Ethics Committee of the State Agency for Nature, Environment and Consumer Protection (LANUV, North Rhine-Westphalia, Germany; Reference number: 84-02.04.2022.A006). All efforts were made to minimize animal suffering and to comply with ethical standards. The study was reported in accordance with the Animal Research: Reporting of In Vivo Experiments (ARRIVE) guidelines [20]. As part of pilot experiments which focused on establishing optimized nerve size conditions, 17-week-old male and female lean mice were euthanized and tissue samples were collected in accordance with the guidelines of Sect. 4 of the German Animal Welfare Act concerning euthanasia and tissue removal. For subsequent experiments, male C57BLKS/J (BKS) (Strain: 683; *n* = 12) and BKS.Cg-Dock7^m^+/+Lepr^db^J (db/db) (Strain: 607; *n* = 12) mice were purchased from the Jackson Laboratories (via Charles River as European distributor) with 5 weeks of age. From 5 weeks of age, mice received a standard diet (*Research Diets*, New Brunswick, USA; Product No: D24032602) consisting of 9 kcal% fat, 71 kcal% carbohydrates and 20 kcal% protein until 16 weeks of age. Two mice were housed per Makrolon type III cage with nesting material provided, and had *ad libitum* access to water and food at a temperature of 22 °C and a 12-hour light-dark cycle. Mice were kept and monitored in accordance with the National Institutes of Health guidelines for the use and care of laboratory animals. Humane endpoints were established to minimize animal suffering. Animals were monitored daily for general health, activity, and signs of pain or distress. Any animal showing severe or persistent signs of illness, > 20% body weight loss, or significant behavioral changes would have been euthanized immediately. The number of animals was determined through a priori power analysis to ensure sufficient statistical power while minimizing animal use. All final experiments and subsequent data analyses were conducted with experimenters blinded to the group allocation. Inclusion and exclusion criteria for animals were not defined a priori. All animals were included in the final analysis, with the exception of one db/db mouse that died in the course of the study, which reduced the sample size in the affected group.

### Blood sample and body weight analysis

Blood glucose concentrations and body weight were assessed at the beginning (week 6) and end (week 15) of the study. Blood samples were collected from the tail tip of unfasted mice. Blood glucose concentrations were measured using a glucometer (*Contour*; Bayer, Leverkusen, Germany), with a detection limit of 33.3 mM. Hyperglycemia was defined as random-fed blood glucose levels exceeding 16.7 mM. This level corresponds to the renal threshold in mice, beyond which the capacity of the kidney to reabsorb glucose is surpassed [21]. Body weight was measured with an electronic scale (*Sartorius*, Göttingen, Germany).

### Isolation of murine sciatic nerves

Mice were fasted for 6 h before being sacrificed by decapitation. To minimize fur contamination, they were rinsed with 70% ethanol and thereafter positioned with their dorsal region facing upwards. Immediately afterward, the skin and muscle tissue surrounding the leg and lumbar region were removed to expose the sciatic nerves. The left and right sciatic nerves were then carefully excised at the knee joint and vertebral column and placed in ice-cooled Seahorse assay medium (Supplementary Table 2). The sciatic nerves were subsequently cut perpendicular to its longitudinal axis into similar-sized fragments, i.e., five 1.5 mm or two 3.0 mm fragments in our pilot experiments. In the study comparing BKS and db/db mice, a total of ten 1.5 mm fragments — five from each sciatic nerve — was used. After isolation, each fragment was transferred into an individual well of a Seahorse XFe96 spheroid plate (*Agilent Technologies*, Santa Clara, CA, USA), which were prefilled with 180 µL of ice-cooled Seahorse assay medium (Supplementary Table 2).

### Extracellular flux analysis

Real-time metabolic analysis of sciatic nerve fragments was performed using a XFe96 Extracellular Flux Analyzer (*Agilent Technologies*). After isolation, oxygen consumption rates (OCR) and extracellular acidification rates (ECAR) were recorded five times at 6 min intervals before subsequent injections with 5 µM oligomycin A (Omy), 2.5 µM carbonyl cyanide 4-(trifluoromethoxy)phenylhydrazone (FCCP), and 5 µM of rotenone (Rot) and 5 µM antimycin A (AA). After each injection, changes in OCR and ECAR were recorded eight times at 6 min intervals. All compounds were obtained from Sigma Aldrich, dissolved in dimethyl sulfoxide (DMSO), stored as 5 mM aliquots at -20 °C, and diluted in Seahorse assay medium (Supplementary Table 2) to the respective concentrations prior to injection. After the assay, each individual trace was examined for an appropriate inhibitor response using the web-based Agilent Analytics tool (*Agilent Technologies*). Traces showing aberrant behavior, such as lack of response to inhibitors, were excluded from analysis. The remaining extracellular flux data were baseline-adjusted for each experimental group. The baseline-adjusted OCR and ECAR values were used to calculate parameters of mitochondrial respiratory function using the metrics of the mitostress test as follows [17]:

- Non-mitochondrial respiration (NMR) (pmol O₂/min) = minimal OCR after injection with Rot/AA.
- Basal respiration (pmol O₂/min) = last OCR before injection with Omy – NMR,
- ATP production (pmol O₂/min) = last OCR before injection with Omy – minimal OCR after injection with Omy,
- Maximal respiration (pmol O₂/min) = maximal OCR after injection with FCCP – NMR,
- Proton leak (pmol O₂/min) = minimal OCR after injection with Omy – NMR,
- Spare respiratory capacity (%) = maximal respiration / basal respiration, and.
- Coupling efficiency (%) = ATP production / basal respiration.

### Mitochondrial toxicity test

Besides the aforementioned mitostress test metrics, we calculated additional metrics based on the so-called mitochondrial toxicity test [22]. To allow these calculations, one well containing a 1.5 mm sciatic nerve fragment from either the left or right sciatic nerve from each mouse was pretreated with 5 µm Rot/AA before the start of the assay. Then, the changes in OCR were recorded in response to Omy, FCCP and Rot/AA injections as described under ‘extracellular flux analysis’. The baseline-adjusted traces for nerve fragments from all mice were averaged and served as a ‘negative reference line’. The positive reference line was defined as the average from the baseline-adjusted traces collected in nerve fragments from the BKS mice. Using these reference lines, we calculated the followed metrics for each trace as follows:

- Mitochondrial toxicity index for the FCCP response (MTI_F_) = [maximal OCR after FCCP injection from test sample – maximal OCR after FCCP injection from positive reference line] / [maximal OCR after FCCP injection from positive reference line – minimal OCR after FCCP injection from negative reference line],
- Mitochondrial toxicity index for uncoupling (MTI_U_) = [maximal OCR of the last 6 readings after Omy injection from test sample – minimal OCR after Omy injection from the positive reference line] / [maximal OCR after FCCP injection from positive reference line – minimal OCR after Omy injection from the positive reference line],
- z-score for inhibition of basal OCR (z-score basal) = [last OCR before injection with Omy from the test sample – mean basal OCR from the positive reference line] / [standard deviation of the basal OCR from the positive reference line],
- z-score for inhibition of the maximal OCR (z-score FCCP) = [maximal OCR after FCCP injection from test sample – mean maximal FCCP injection from the positive reference line] / [standard deviation from the maximal OCR after FCCP injection from the positive reference line].

### Normalization of extracellular flux data to the mitochondrial enrichment factor

Extracellular flux data were normalized to mitochondrial protein content rather than total protein or tissue weight, as mitochondrial abundance can vary substantially between lean BKS and diabetic db/db mice. To estimate the mitochondrial protein content, label-free quantitative proteomic profiling of sciatic nerve samples was performed in triplicate for each mouse using high-resolution mass spectrometry (MS). A detailed description of the MS-based proteome analysis, including sample preparation, MS acquisition settings, MS data analysis, is provided in the Supplementary Information. The MitoCarta 3.0 database was used to define mitochondrial proteins within the proteomic dataset. For each sample, a mitochondrial enrichment factor (MEF) was calculated as the ratio of MitoCarta 3.0-annotated mitochondrial proteins to total protein abundance. The resulting MEF was then used to normalize the corresponding aforementioned extracellular flux data, enabling mitochondrial respiration parameters to be expressed relative to mitochondrial protein content.

### Citrate Synthase (CS) activity and total protein content

Five 1.5 mm sciatic nerve segments per mouse were pooled for analysis. Tissue homogenization was performed in 65µL of lysis buffer (Supplementary Table 2) and involved mechanical disruption using a pistil, passage through a 26-gauge insulin syringe (10 strokes), and sonication (two cycles; interval: 0.09 s; duration: 10 s; *Sonoplus*,* Bandelin*). After centrifugation at 900 × g for 10 min at 4 °C, the supernatant was collected for subsequent analyses. Total protein concentration was determined using the BCA Protein Assay Kit (*Thermo Fisher*, Waltham, MA, USA). CS activity was measured using the Citrate Synthase Activity Assay Kit (*Sigma-Aldrich*, Steinheim, Germany) and normalized to the protein content of each sample.

### Statistics

Data are reported as mean ± standard deviation (SD). Significant differences between groups were determined by unpaired two-tailed Student’s t-test or by two-way ANOVA with Šídák’s *post hoc* multiple comparison test. *P* values < 0.05 were considered statistically significant. All statistical analyses were performed using the GraphPad Prism 10 software (Software-Version 10.4.2; GraphPad Software, San Diego, CA, USA).

### Data availability

The datasets generated and/or analysed during the current study are available from the corresponding author’s on reasonable request.

## Results

### Optimizing mitochondrial respiration assays in murine sciatic nerve fragments: impact of fragment length on respiratory function

**Fig. 1:**
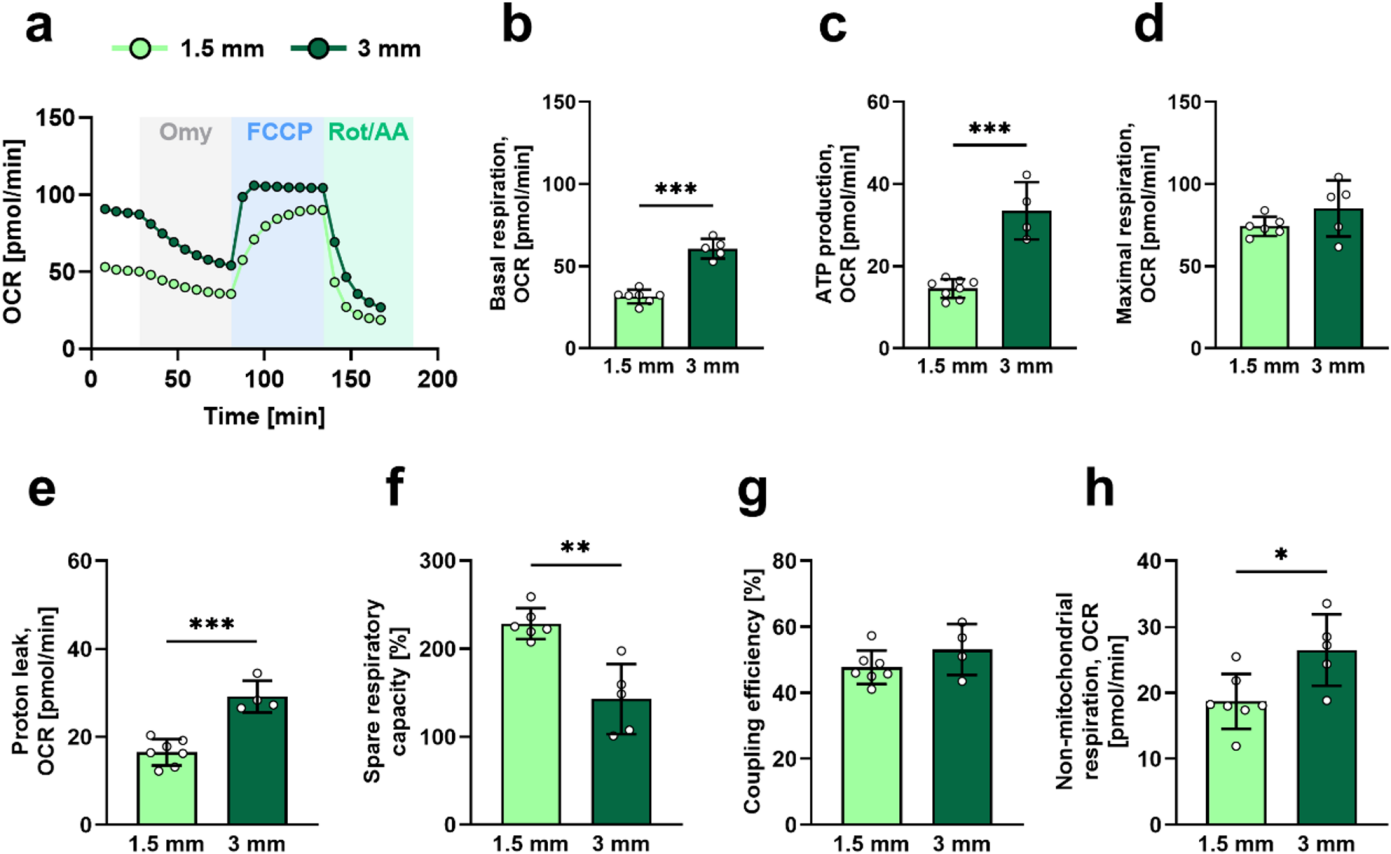
Impact of sciatic nerve fragment size on determinants of mitochondrial respiratory function in murine sciatic nerves. **a** Effect of Omy, FCCP and Rot/AA on OCR in 1.5 mm (*n* = 8) and 3 mm (*n* = 5) sciatic nerve fragments. The bar graphs show the quantification of basal respiration (**b**), ATP production (**c**), maximal respiration (**d**), proton leak (**e**), spare respiratory capacity (**f**), coupling efficiency (**g**), and non-mitochondrial respiratory capacity (**h**). Each dot represents an individual measurement of sciatic nerve fragments collected from two mice. The data are expressed as mean ± SD. Differences between the two groups were assessed using an unpaired *t*-test. *, **, and *** indicate a *p*-value < 0.05, < 0.01, and < 0.001 between indicated groups, respectively

Several pilot experiments were conducted in lean, non-diabetic mice based on established protocols [16, 18, 23] to enable optimal quantification of mitochondrial respiratory function in isolated murine sciatic nerves. These experiments focused on optimizing the assay parameters such as plate type, nerve fragment size, and the inhibitor response. In our hands, the use of standard plates for the assay resulted in inconsistent OCR measurements and non-reproducible responses to mitochondrial inhibitors due to fragment flotation, despite the use of various coating agents (data not shown). The use spheroid 96-well plates effectively overcame these limitations. Figure 1a illustrates the impact of sequential injections of the ATP synthase inhibitor Omy, the uncoupling agent FCCP, and the complex I and III inhibitors Rot and AA on isolated sciatic nerve fragments obtained from lean, non-diabetic mice. Fragments measuring 1.5 and 3 mm displayed basal respiratory rates of 31.4 ± 4.1 and 60.7 ± 6.0 pmol O₂/min, respectively (Fig. 1b). Following Omy injection, mitochondrial ATP production was 14.6 ± 2.3 and 33.5 ± 7.0 pmol O₂/min in 1.5 mm and 3 mm sciatic nerve fragments, respectively (Fig. 1c).

After injection with FCCP, maximal respiration increased to 74.3 ± 5.8 pmol O₂/min and 85.1 ± 17.1 pmol O₂/min in 1.5 mm and 3 mm fragments, respectively (Fig. 1d). The larger fragments exhibited higher values for these parameters but also displayed signs of mitochondrial stress. This was indicated by increased proton leakage (29.2 ± 3.6 versus 16.5 ± 3.0 pmol O₂/min; Fig. 1e) and reduced spare respiratory capacity (143% ± 39.7% versus 228% ± 17.7%; Fig. 1f) in 3 mm versus 1.5 mm fragments. Fragment size did not affect coupling efficiency (48% ± 5.1% versus 53% ± 7.7%; Fig. 1g), whereas non-mitochondrial respiration was higher in the 3 mm fragments (26.5 ± 5.4 versus 18.7 ± 4.2 pmol O₂/min; Fig. 1h). Therefore, 1.5 mm fragments were used for all subsequent experiments.

### Impaired mitochondrial respiration in sciatic nerves of diabetic db/db mice compared to lean BKS controls

**Fig. 2:**
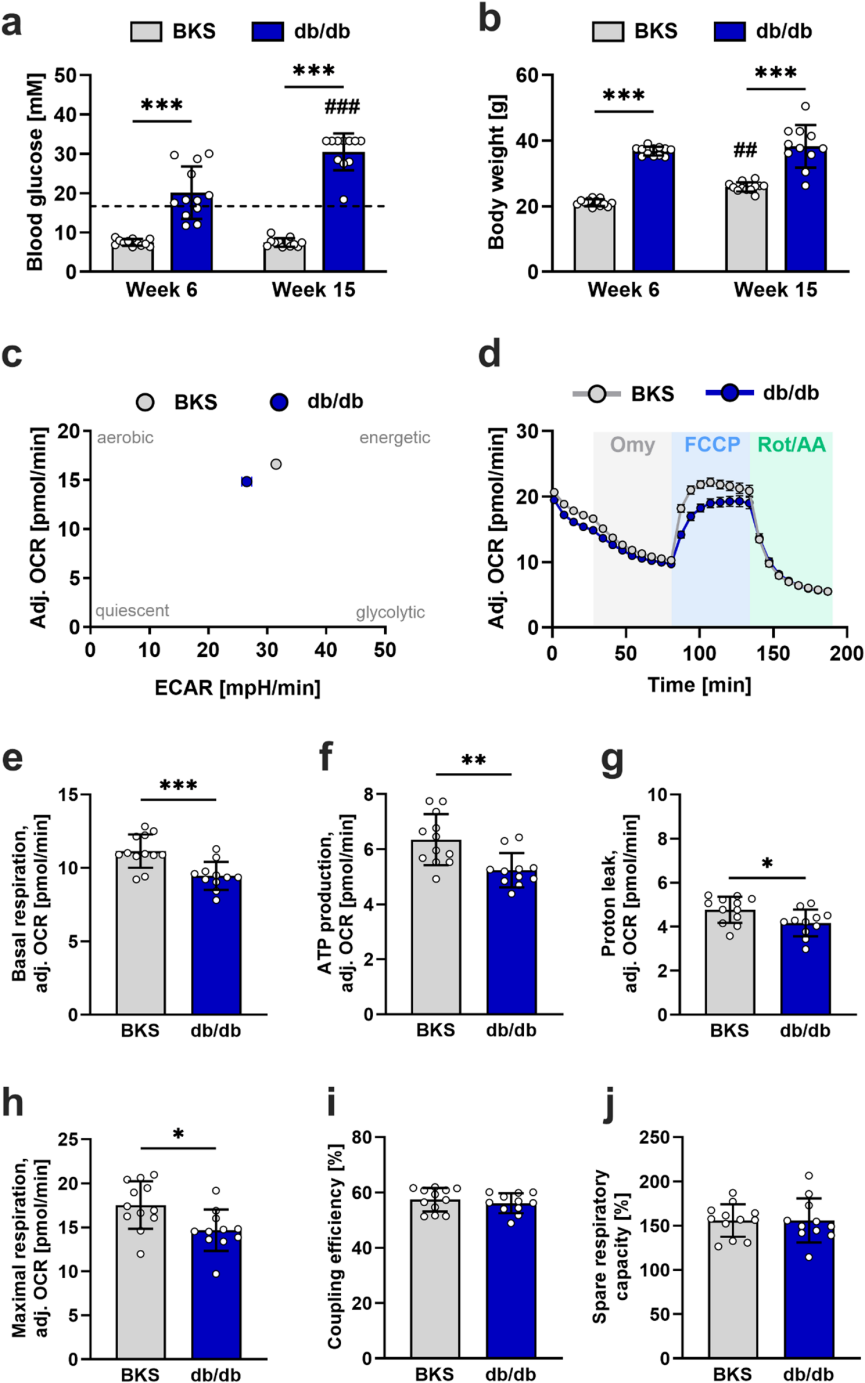
Mitochondrial respiratory function in sciatic nerves from BKS and diabetic db/db mice. The bar graphs show blood glucose levels (**a**) and body weight (**b**) of BKS (grey bars, n=12) and db/db mice (blue bars, n=11-12) at six and 15 weeks of age. The dashed line indicates the renal threshold for glucose reabsorption (16.7 mM), above which hyperglycemia was defined. **c** Energy map for basal adjusted OCR and ECAR in BKS and db/db mice. **d** Effect of Omy, FCCP and Rot/AA on adjusted OCR in BKS and db/db mice. The bar graphs show the quantification of basal respiration (**e**), ATP production (**f**), proton leak (**g**), maximal respiration (**h**), coupling efficiency (**i**) and spare respiratory capacity (**j**). The dots represent the mean values collected for the nerve fragments obtained in each mouse. The data are expressed as mean ± SD. Differences between the two groups were assessed using an unpaired t-test. *, **, and *** indicate a p-value <0.05, <0.01, and <0.001 between indicated groups, respectively. ## and ### indicate a p-value <0.01 and <0.001, respectively, between week 6 and week 15

To evaluate whether diabetes affects peripheral nerve mitochondrial respiration, we compared OCR in sciatic nerve fragments from 15-week-old diabetic db/db mice with those from lean BKS control mice using a Seahorse XFe96 extracellular flux analyzer. At six weeks of age, db/db mice already displayed marked hyperglycemia (20.1 ± 6.6 versus 7.5 ± 0.8 mM; Fig. 2a) and higher body weights (36.8 ± 1.4 versus 21.1 ± 1.0 g; Fig. 2b) compared with BKS mice. By 15 weeks of age, blood glucose levels in db/db mice had increased further (Week 6: 20.1 ± 6.6 mM; Week 15: 30.5 ± 4.7 mM), whereas BKS controls remained normoglycemic (Week 6: 7.5 ± 0.8 mM; Week 15: 7.5 ± 1.1 mM) (Fig. 2a). OCR recordings showed no differences between fragments isolated from the left or right sciatic nerve in either BKS or db/db mice (Supplementary Fig. 1a, b). Therefore, the data from the left and right nerves were pooled for each animal in subsequent analyses. Prior to quantifying determinants of mitochondrial function, OCR recordings were normalized to the MEF, which was derived from proteomic analyses of approximately 3 mm fragments of the corresponding sciatic nerve [24], and baseline-adjusted for each group. Notably, neither the mitochondrial enrichment factor nor its defining components, namely the number and the abundance of total and mitochondrial peptides, differed between BKS and db/db mice (Supplementary Fig. 2a, Supplementary Table 3). In addition to the MEF, commonly used normalization factors, including CS activity and total protein concentration, were assessed (Supplementary Fig. 2b, c). These measures likewise showed no genotype dependent differences but exhibited higher inter sample variability compared with the MEF and were therefore not used for normalization in subsequent analyses.

Figure 2c shows the energy map, which indicates lower adjusted OCR (14.8 ± 0.2 versus 16.6 ± 0.2 pmol O₂/min) and ECAR (26.5 ± 0.8 versus 31.5 ± 0.4 mpH/min) in the sciatic nerves of db/db mice compared to BKS mice prior to injection with Omy. Following the injections with Omy and FCCP, the adjusted OCR in sciatic nerves from db/db mice remained lower than in controls (Figure 2d). Consequently, basal respiration (9.5 ± 1.0 versus 11.2 ± 1.1 pmol O₂/min; Figure 2e), ATP production (5.2 ± 0.6 versus 6.4 ± 0.9 pmol O₂/min; Figure 2f), proton leakage (4.2 ± 0.6 versus 4.8 ± 0.6 pmol O₂/min; Figure 2g), and maximal respiration (14.7 ± 2.3 versus 17.5 ± 2.7 pmol O₂/min; Figure 2h) decreased in sciatic nerves from db/db mice compared to BKS mice. There were no changes in coupling efficiency (56.1 ± 3.6 versus 57.4 ± 4.2%; Figure 2i), spare respiratory capacity (155.8 ± 24.8 versus 155.8 ± 18.3%; Figure 2j), or non-mitochondrial respiration (data not shown) between the mouse strains.

### Implementation of novel mitochondrial toxicity metrics in murine sciatic nerve OCR analysis

**Fig. 3:**
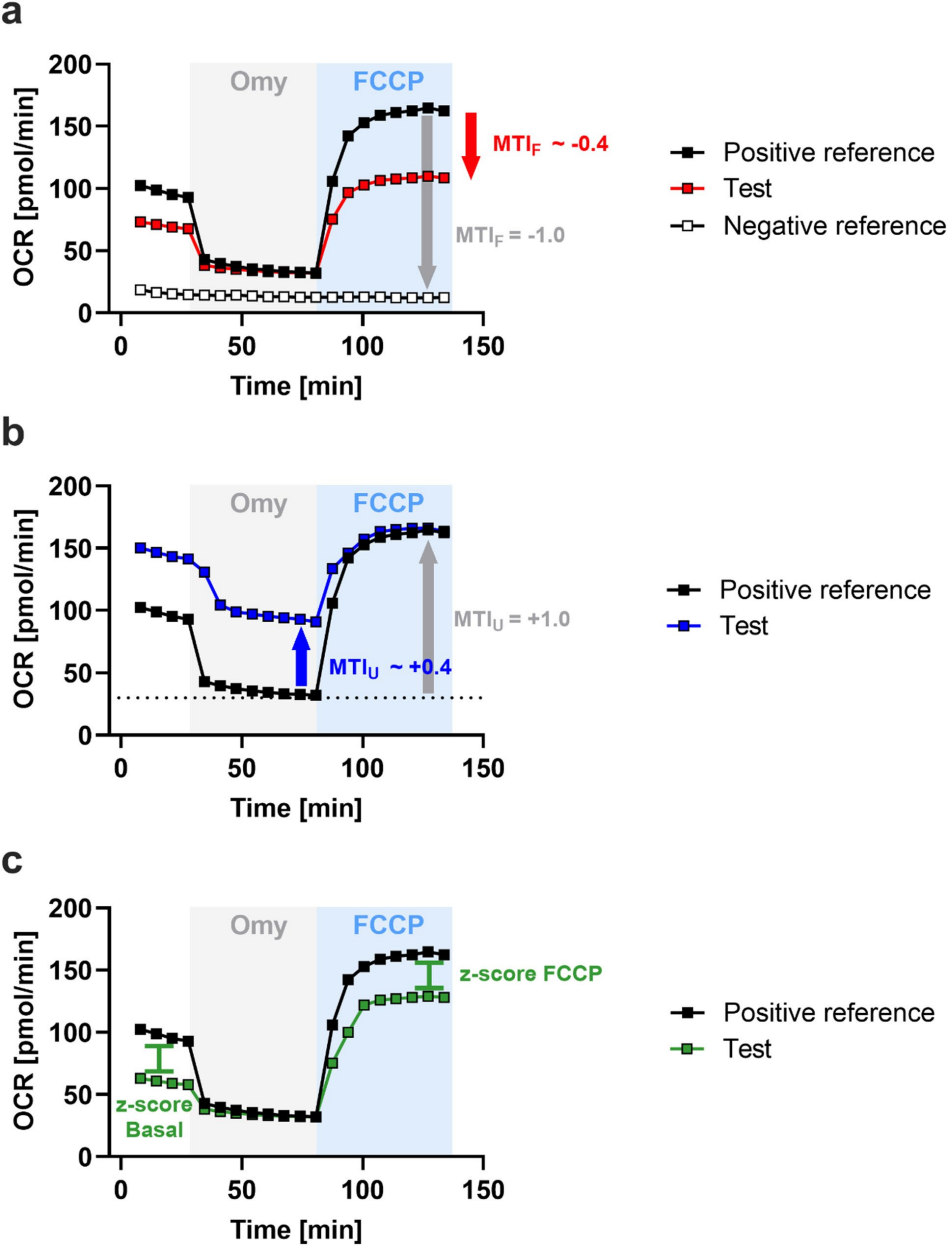
Calculation of new mitochondrial activity metrics. **a** MTI_F_, (**b**) MTI_U_, and (**c**) z-score basal and FCCP. The OCR trace of BKS mice served as the positive reference line, representing maximal activity, while the Rot/AA-treated trace was used as the negative reference line, reflecting complete inhibition

Recently, new metrics were described that enable more reliable and straightforward detection and evaluation of drug-induced mitochondrial toxicity in cultured cells [22]. These metrics compare the response of a test group with two reference groups: A positive control group displaying maximal activity and a negative control treated with Rot/AA prior to the assay. To implement these metrics in our experimental setup, we recorded OCR from one nerve segment per mouse in the presence of Rot/AA. This trace served as the negative reference line (Fig. 3a). The OCR trace obtained from sciatic nerves of BKS mice was used as the positive reference line. Based on these reference lines, we calculated three indexes: (i) the mitochondrial toxicity index for the FCCP response (MTI_F_), (ii) the mitochondrial toxicity index for uncoupling (MTI_U_), and (iii) z-scores for the inhibition of basal OCR and maximal OCR (Fig. 3a-c).

### Reduced mitochondrial toxicity indices and respiratory function in sciatic nerves of diabetic db/db mice

**Fig. 4:**
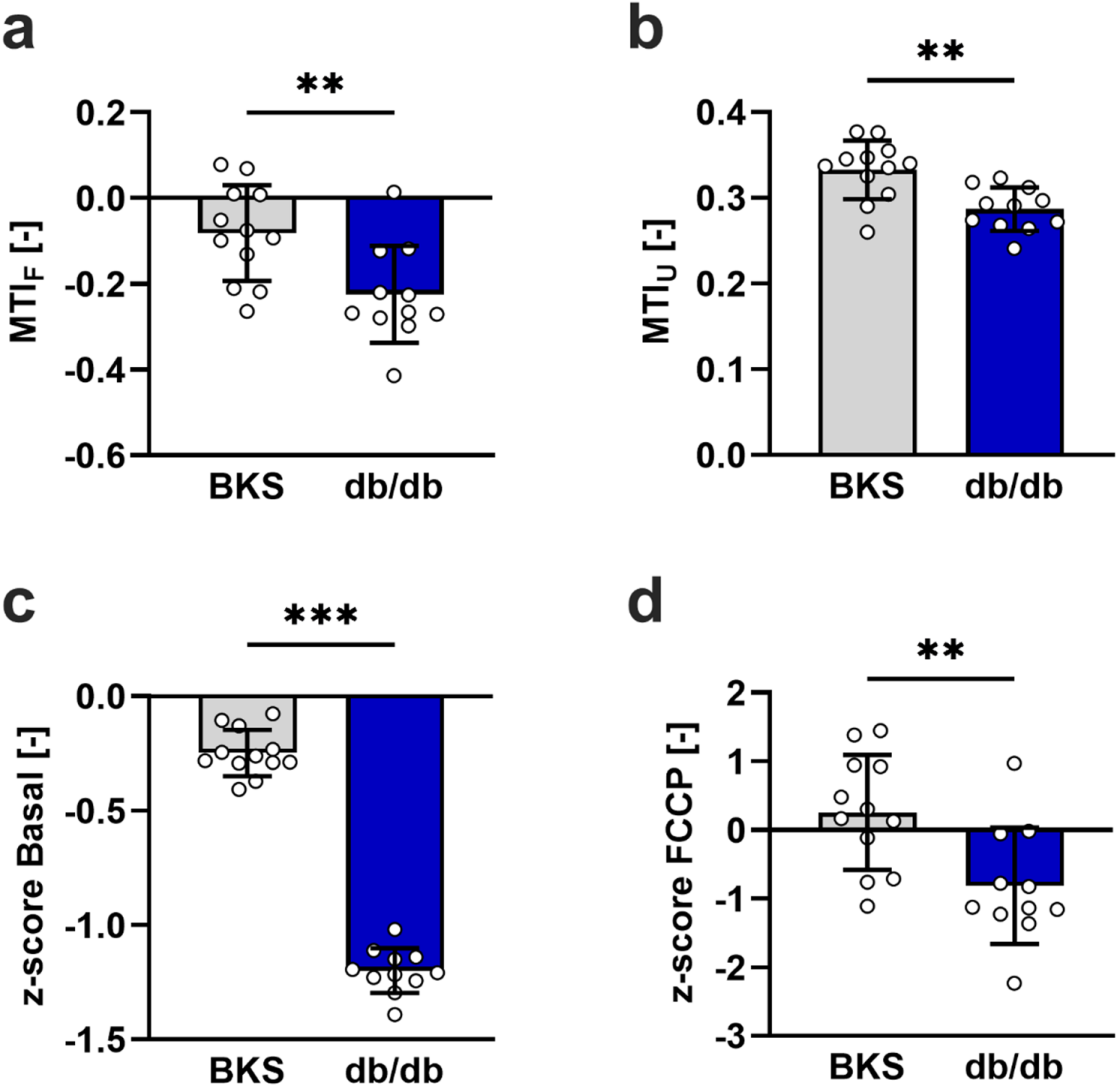
Quantification of mitochondrial functionality in sciatic nerves from BKS and diabetic db/db mice using novel metrics. Bar graphs showing the quantification of the MTI_F_ (**a**), MTI_U_ (**b**), and z-scores for changes in basal (**c**) and maximal OCR after FCCP injection (**d**). The dots represent the mean scores for the sciatic nerve fragments obtained in each mouse. The data are expressed as mean ± SD. Differences between the two groups were assessed using an unpaired *t*-test. ** and *** indicate a *p*-value < 0.01 and < 0.001, respectively

Figure 4a shows that the sciatic nerves from db/db mice had a lower MTI_F_ than the BKS group (-0.22 ± 0.1 versus − 0.08 ± 0.1). The MTI_F_ was used to quantify the change in the FCCP response of the sciatic nerves from db/db mice relative to the established reference lines. The FCCP response in the BKS group was considered to display 0% inhibition. The minimal response (100% inhibition) was derived from the trace recorded in the presence of Rot/AA (Fig. 3a). The MTI_U_ quantifies changes in coupling between basal respiration and ATP production. We calculated this index by considering the maximal OCR observed after FCCP injection in the BKS reference group as 100% uncoupling and the minimal OCR after Omy in this group as 0% uncoupling (Fig. 3b). Figure 4b shows that db/db mice had a lower MTI_U_ than the BKS group (0.29 ± 0.03 versus 0.33 ± 0.03). Decreased mitochondrial activity, which is caused by the direct inhibition of oxidative phosphorylation (OxPhos) machinery (including ATP synthase), usually results in lower basal respiration without altering the OCR response to subsequent mitochondrial inhibitors. To determine changes in basal mitochondrial respiratory activity, we calculated a z-score by comparing the basal OCR of db/db mice to the mean basal OCR of BKS controls (Fig. 3c). As shown in Fig. 4c, db/db mice exhibited a pronounced reduction in the z-score for basal OCR (-1.2 ± 0.1 versus − 0.25 ± 0.1). To further distinguish between the inhibition of the OxPhos machinery and other types of mitochondrial toxicity, a z-score was calculated for the maximal response to FCCP in both strains (Fig. 3c). Figure 4d shows that this z-score was also lower in db/db mice (-0.81 ± 0.8 versus 0.26 ± 0.8).

## Discussion

In this study, we present an optimized ex vivo protocol for assessing mitochondrial respiratory function in isolated murine sciatic nerves. This protocol enables robust characterization of abnormal mitochondrial function in the context of diabetic polyneuropathy. Analysing multiple 1.5 mm nerve fragments per mouse and averaging data across technical replicates and the left and right nerves minimizes the impact of metabolic heterogeneity and enhances the precision of mitochondrial readouts. In addition, the use of spheroid plates prevented fragment flotation during the assay even in the absence of coating agents, which in turn may affect mitochondrial inhibitor concentrations by quenching. Therefore, we consider this approach as a significant methodological advance over previous methods that evaluate the entire nerve as a single unit, which increases variability and limits the ability to detect subtle bioenergetic differences.

In addition, we applied the proteomics-based MEF, which is defined as the ratio between the total abundance of mitochondrial peptides and the total abundance of all peptides in each nerve fragment, for normalization of the flux data. This previously reported index [24] displayed lower variations among the nerve fragments used in this study compared with other commonly used normalization approaches, such as total protein content or CS activity. Nevertheless, these other normalization approaches can replace the MEF in the workflow when proteomics-based analysis are not available. Our study further introduces novel dimensionless mitochondrial toxicity indices (MTI_F_ and MTI_U_) and z-scores for basal and maximal OCR as tools to improve the interpretability of extracellular flux data in peripheral nerves. These reference-based metrics overcome key limitations of conventional parameters, such as spare respiratory capacity, which is often ambiguous when the FCCP response is attenuated or the baseline OCR and response to Rot/AA are variable [22]. In our study, although there was no difference in spare respiratory capacity between the BKS and db/db groups, both MTI_F_ and MTI_U_ showed impairments in FCCP-induced uncoupling and in coupling between basal and ATP-linked respiration. This discrepancy underscores how traditional readouts can underrepresent mitochondrial impairments in models of chronic metabolic stress and highlights the value of incorporating positive and negative reference lines to normalize responses. Application of these novel metrics revealed several bioenergetic abnormalities in the sciatic nerves of diabetic db/db mice. First, reduced MTI_F_ in db/db nerves indicates a blunted response to mitochondrial uncoupling reflective of compromised electron transport chain capacity. Second, the lower MTI_U_ indicates decreased ATP production efficiency relative to basal respiration, which is consistent with early-stage OxPhos dysfunction. Third, the marked decrease in z-scores for basal and maximal OCR indicates generalized mitochondrial impairment rather than selective inhibition of complexes. Together, these functional data support prior histological and biochemical reports of mitochondrial structural abnormalities [25], increased reactive oxygen species [26, 27], and reduced mitochondrial biogenesis signalling [28] in the peripheral nerves of diabetic rodents. They also provide direct evidence of compromised bioenergetic reserves and electron transport chain integrity.

To our knowledge, this is the first study to combine spatially resolved, high‑replicate mitochondrial analysis of sciatic nerve segments with mitotoxicity metrics in a diabetic model. Such methodological innovation allowed us to uncover previously underappreciated aspects of abnormal mitochondrial function in the db/db mouse model of type 2 diabetes, strengthening its utility for preclinical evaluation of mitochondria‑targeted therapies. For instance, interventions aimed at enhancing PGC‑1α–mediated biogenesis [28] or at reducing mitochondrial calcium overload [29] could be directly screened using our assay.

Peripheral nerve dysfunction, especially in cases of diabetic polyneuropathy, is challenging to model and quantify in animals. This is partly due to the limited sensitivity of traditional functional assessments. Although behavioural, electrophysiological, and histopathological measures are the current standard, they often fail to detect early or subtle impairments, especially those associated with abnormal mitochondrial function. Given the emerging role of mitochondrial bioenergetic failure in the pathogenesis of peripheral neuropathies [30], there is a critical need for reliable approaches in disease models that can directly assess mitochondrial function within nerve tissue. Our optimized implementation of ex vivo mitochondrial respiration analysis in isolated peripheral nerves fills this gap by enabling high-resolution interrogation of respiratory capacity and coupling efficiency at different disease stages. This method should enable the detection of early bioenergetic defects before overt axonal degeneration or conduction slowing occurs. Notably, this approach can be applied to various disease models, providing a robust and clinically relevant tool for characterizing the role of mitochondria in peripheral nerve injury. These assays may also serve as sensitive biomarkers for therapeutic response or progression, particularly in early-stage or preclinical disease settings, by providing insight into potential changes in mitochondrial function in the trajectory of nerve dysfunction.

## Conclusion

In conclusion, our findings support the use of refined, fragment-based mitochondrial profiling in peripheral nerves to overcome challenges related to tissue heterogeneity and interpretive ambiguity in conventional assays. The mitotoxicity metrics introduced here improve the reliability and effectiveness of extracellular flux analysis, especially in pathological conditions where traditional parameters may not fully capture bioenergetic defects. These advances together open new avenues for therapeutic screening in diabetic neuropathy and other disorders characterized by abnormal mitochondrial functionality in peripheral nerves.

## Supplementary Information

Supplementary Material 1.

## Data Availability

The datasets generated and/or analysed during the current study are available from the corresponding author’s on reasonable request.
