# Supplementary material for: A novel extracellular flux assay workflow uncovers impaired sciatic nerve mitochondrial respiration in diabetic db/db mice
Source: Cell Commun Signal. 2026 Jan 21;24:58. doi: 10.1186/s12964-026-02667-9 (PMC12849079; doi:10.1186/s12964-026-02667-9)
Supplement: Supplementary file 1 — Supplementary Material 1. [file 12964_2026_2667_MOESM1_ESM.docx]

**Supplementary Material**

**Supplementary Figures**


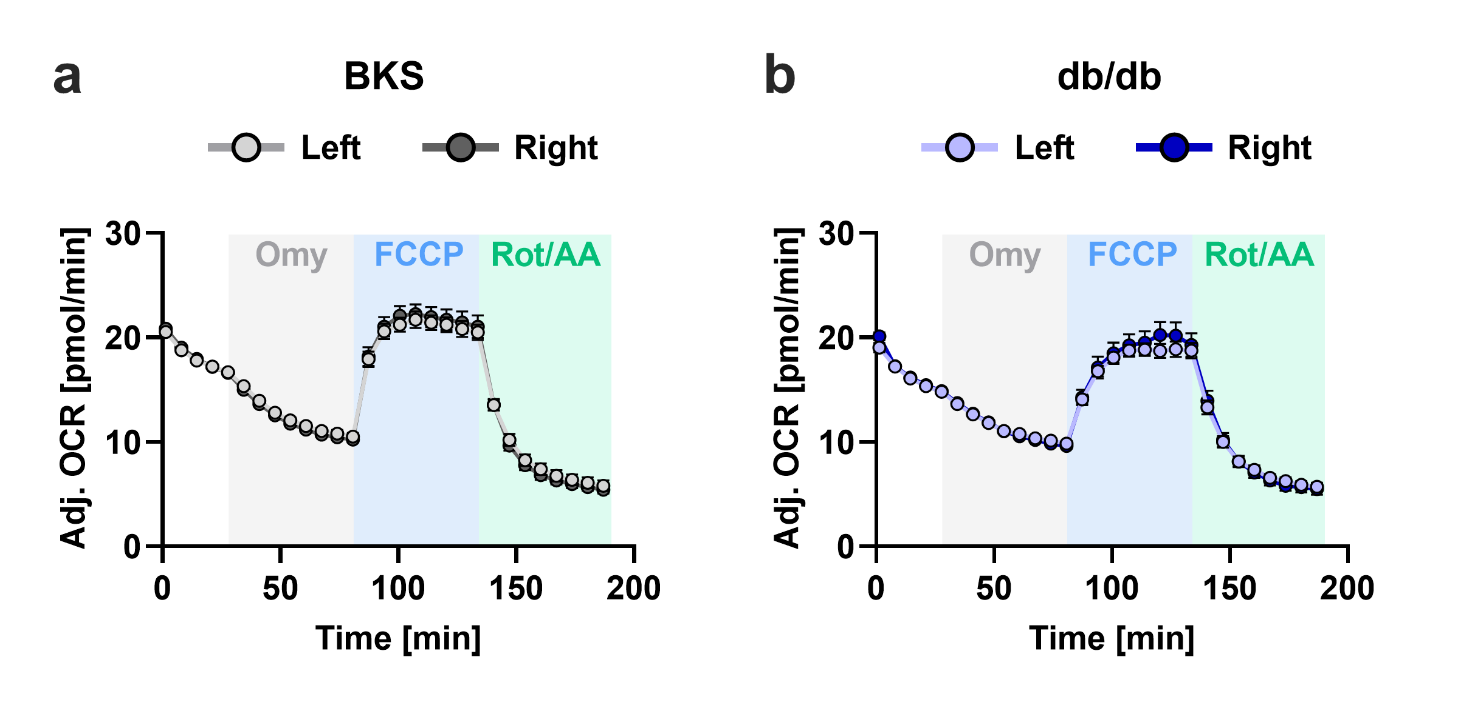


**Supplementary Figure 1.** Impact of oligomycin A (Omy), carbonyl cyanide p-trifluoromethoxyphenylhydrazone (FCCP) and rotenone (Rot)/antimycin A (AA) on oxygen consumption rate (OCR) in fragments isolated from the left and right sciatic nerves from C57BLKS/J (BKS) (n=12) (**a**) and db/db mice (n=11) (**b**). For each mouse the data obtained in the fragments of the left and right nerve were averaged. The plot shows the mean ± standard deviation (SD) for the adjusted OCR values collected in each mouse.


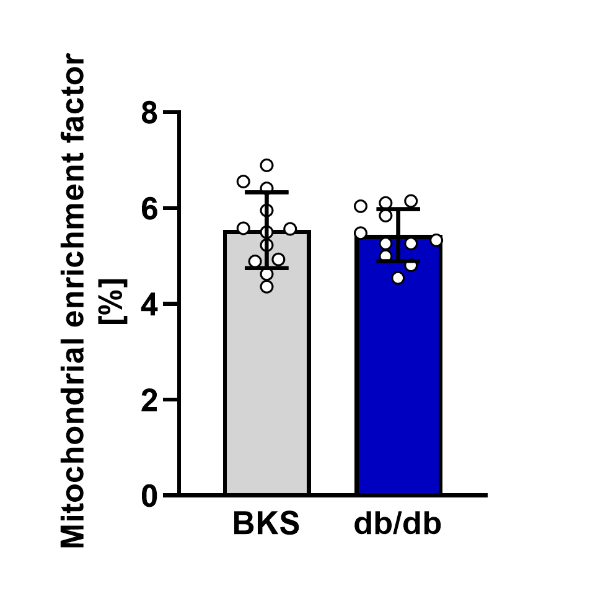


**Supplementary Figure 2.** Mitochondrial enrichment factor (MEF) determined from proteomic analyses of sciatic nerve fragments from BKS (n=12) and db/db mice (n=11). The MEF was calculated as the ratio of MitoCarta3.0-annotated mitochondrial proteins to total protein abundance. MEF values were subsequently used to normalize the corresponding oxygen consumption rate (OCR) recordings. Data are presented as mean ± standard deviation (SD). Differences between the two groups were assessed using an unpaired *t*-test.

**Supplementary Methods**

**Sample preparation for mass spectrometry (MS)-based proteome analyses of sciatic nerves**

Approximately 3 mm segments of sciatic nerves were collected from lean BKS (n = 12) and diabetic db/db (n = 11) mice and immediately snap-frozen in liquid nitrogen. Tissue homogenization was performed in a denaturing lysis buffer composed of 100 mM Tris-HCl, 4% SDS, and 100 mM DTT, supplemented with protease and phosphatase inhibitor cocktails (cOmplete™, Roche; PhosSTOP™, Merck). The homogenization process included mechanical disruption via pistil grinding, stroking through a 26-gauge insulin syringe (10 times), and sonication (two times pulse 0.09 s_10 s; Sonoplus, Bandelin). After centrifugation at 75,000 x g for 30 min at 4°C, supernatants were transferred to new tubes and protein concentrations were quantified by direct photometric measurements using a NanoDrop™ spectrophotometer (ThermoFisher Scientific). For proteolytic digestion, approximately 20 µg protein from each sample was processed using µS-Trap columns (ProtiFi), with a Trypsin/Lys-C mixture (Promega, Cat. No.: V5071) in a 1:25 (*w*/*w*) enzyme/protein ratio. Lyophilized peptides were reconstituted in 1% trifluoroacetic acid (TFA, *v*/*v*), and peptide concentrations were measured with the Pierce™ Quantitative Colorimetric Peptide Assay (ThermoFisher Scientific, Cat. No.: 23275). For liquid chromatography (LC)-mass spectrometry (MS)/MS analysis, 400 ng of peptides per sample was injected in technical triplicates onto a Vanquish Neo UHPLC system (Thermo Fisher Scientific) coupled to an Orbitrap Fusion™ Lumos™ mass spectrometer via a Nanospray Flex™ ion source and equipped with a high-field asymmetric waveform ion mobility spectrometry (FAIMS Pro) interface (Thermo Fisher Scientific), operating in data-dependent acquisition (DDA) mode. Peptides were initially trapped and desalted on an Acclaim™ PepMap™ C18 pre-column (75 μm inner diameter, 2 cm length; ThermoFisher Scientific), followed by being separated on an Aurora Series C18 analytical column (AUR2-25075C18A, 25 cm × 75 μm C18 1.6 µm; IonOpticks) at a flow rate of 300 nL/min. Peptides were chromatographically separated using a three-phase linear gradient with mobile phase A (0.1% formic acid) and mobile phase B (80% acetonitrile, 0.1% formic acid) over 120 minutes: 2–19% mobile phase B for 72 min, 19–29% mobile phase B for 28 min, 29–41% mobile phase B for 20 min, and finally increased to 95% mobile phase B in 1 min. Full MS scans were acquired utilizing two FAIMS compensation voltages (CV) of -45 (1.8 s cycle time) and -65 (1.2 s cycle time) each at a resolution of 120,000 (at 200 m/z) over an m/z range of 350-1,600. Automatic gain control (AGC) target value were set to 4e^5^ and injection time were adjusted automatically. Precursors with charge states between two and seven were selected for fragmentation, applying a dynamic exclusion of 30 s. MS/MS spectra were acquired at a resolution of 30,000 using higher-energy collisional dissociation (HCD) with a normalized collision energy of 30%. Fragment ions were isolated with a 3.6 m/z window, and AGC target values of 5e^4^, with maximal injection time of 54 ms.

**Analyses of MS-based data**

Raw MS data were analyzed using the Proteome Discoverer™ 3.2 software (Thermo Fisher Scientific). Spectral recalibration was performed using the Spectrum RC node against the reviewed SwissProt *Mus musculus* ((sp_canonical TaxID=10090) - [Release=407] (v2025-02-05)). For label-free quantification, the Minora Feature Detector node was used with default settings, including a minimum trace length of five, a maximum delta retention time of 0.2 min for isotope pattern multiplets, and feature to ID linking was restricted to high-confidence peptide-spectrum matches (PSMs). Protein identification was performed using the Chimerys search engine (Prediction model inferys_4.7.0_fragmentation) against the reviewed SwissProt *Mus musculus* database including isoforms ((sp_incl_isoforms TaxID=10090) - [Release=407] (v2025-02-05)), supplemented with an in-house contaminant database. Trypsin was specified as the proteolytic enzyme, allowing up to two missed cleavages. Carbamidomethylation of cysteine was set as a static modification, while methionine oxidation was set as a dynamic modification. For Protein grouping strict parsimony principle were applied. Label-free quantification was performed on precursor intensity present in min. 20% of the replicates. Protein ratios were calculated pairwise ratio based (t-test, background based). The MS data were filtered to include (1) “master proteins” (proteins with the longest sequence were selected as master, when multiple proteins had the same score, number of PSMs, and matched peptides), (2) proteins with false discovery rate (FDR) < 0.01 (i.e., high confidence) in protein FDR confidence combined, (3) proteins with at least one “unique peptide”, (4) proteins annotated with “species map” for Mus musculus, and (5) proteins identified with high confidence in at least six samples. Mitochondrial proteins were identified using the murine MitoCarta3.0 database (<https://www.broadinstitute.org/mitocarta/>), which includes 1,140 genes encoding proteins with strong evidence for mitochondrial localization. A mitochondrial enrichment factor (MEF) was calculated as the ratio of MitoCarta3.0-annotated proteins to total protein abundance and was subsequently used to normalize mitochondrial respiration parameters to mitochondrial protein content.

**Supplementary Tables**

**Supplementary Table 1:** *Chemicals and materials*

| **Compound/Material** | **Manufacturer** | **Product Code** |
| --- | --- | --- |
| Antimycin A | Sigma-Aldrich, St. Louis, MO, USA | A8674 |
| Carbonyl cyanide 4-(trifluoromethoxy)phenylhydrazone (FCCP) | Sigma-Aldrich, St. Louis, MO, USA | C2920 |
| d(+)-Glucose anhydrous | AppliChem, Darmstadt, Germany | A0883,1000 |
| Dimethyl sulfoxide (DMSO) | AppliChem, Darmstadt, Germany | A3672 |
| Ethanol | Sigma-Aldrich, St. Louis, MO, USA | 1.00974 |
| l-Glutamine | Sigma-Aldrich, St. Louis, MO, USA | G5792 |
| Oligomycin A | Sigma-Aldrich, St. Louis, MO, USA | 75351 |
| Rotenone | Sigma-Aldrich, St. Louis, MO, USA | R8875 |
| Seahorse XF DMEM medium (pH 7.4) | Agilent Technologies, Santa Clara, CA, USA | 103575-100 |
| Seahorse XFe96 Extracellular Flux Analyzer | Agilent Technologies, Santa Clara, CA, USA | S7800B |
| Seahorse XFe96 Spheroid FluxPak | Agilent Technologies, Santa Clara, CA, USA | 102905-100 |
| Sodium pyruvate | Sigma-Aldrich, St. Louis, MO, USA | P2256 |

**Supplementary Table 2:** *Buffer*

| **Description** | **Ingredients** |
| --- | --- |
| Seahorse assay medium | Seahorse XF DMEM medium (pH 7.4) supplemented with 2 mM l-glutamine, 1 mM sodium pyruvate and 10 mM  d-glucose |
